# Low-Intensity Pulsed Ultrasound Accelerates Tooth Movement via Activation of the BMP-2 Signaling Pathway

**DOI:** 10.1371/journal.pone.0068926

**Published:** 2013-07-23

**Authors:** Hui Xue, Jun Zheng, Ziping Cui, Xiufeng Bai, Gang Li, Caidi Zhang, Sanhu He, Weihong Li, Shayanne A. Lajud, Yinzhong Duan, Hong Zhou

**Affiliations:** 1 Department of Orthodontics, School of Stomatology, Fourth Military Medical University, Xi'an, China; 2 Department of Dentistry, Hegang People's Hospital, Hegang, China; 3 Department of Oral and Maxillofacial Surgery, Stomatological Hospital of Xi'an Jiaotong University, Xi'an, China; 4 Department of Digestive Diseases, Hegang People's Hospital, Hegang, China; 5 Department of Orthodontics, Stomatological Hospital of Xi'an Jiaotong University, Xi'an, China; 6 Department of Orthodontics, Stomatological Hospital of Wenzhou Medical College, Wenzhou, China; 7 Department of Otorhinolaryngology-Head and Neck Surgery, University of Pennsylvania School of Medicine, Philadelphia, Pennsylvania, United States of America; Boston University Goldman School of Dental Medicine, United States of America

## Abstract

The present study was designed to determine the underlying mechanism of low-intensity pulsed ultrasound (LIPUS) induced alveolar bone remodeling and the role of BMP-2 expression in a rat orthodontic tooth movement model. Orthodontic appliances were placed between the homonymy upper first molars and the upper central incisors in rats under general anesthesia, followed by daily 20-min LIPUS or sham LIPUS treatment beginning at day 0. Tooth movement distances and molecular changes were evaluated at each observation point. *In vitro* and *in vivo* studies were conducted to detect HGF (Hepatocyte growth factor)/Runx2/BMP-2 signaling pathways and receptor activator of NFκB ligand (RANKL) expression by quantitative real time PCR (qRT-PCR), Western blot and immunohistochemistry. At day 3, LIPUS had no effect on the rat orthodontic tooth movement distance and BMP-2-induced alveolar bone remodeling. However, beginning at day 5 and for the following time points, LIPUS significantly increased orthodontic tooth movement distance and BMP-2 signaling pathway and RANKL expression compared with the control group. The qRT-PCR and Western blot data *in vitro* and *in vivo* to study BMP-2 expression were consistent with the immunohistochemistry observations. The present study demonstrates that LIPUS promotes alveolar bone remodeling by stimulating the HGF/Runx2/BMP-2 signaling pathway and RANKL expression in a rat orthodontic tooth movement model, and LIPUS increased BMP-2 expression via Runx2 regulation.

## Introduction

Accelerating the alveolar bone remodeling process and consequently accelerating the rate of tooth movement is highly desirable for orthodontic patients. Methods to stimulate bone remodeling, such as electric stimulation [1], drug injections [2], corticision and alveolar corticotomy [3], [4], [5], [6], [7], Low-energy laser [8] and low-intensity pulsed ultrasound (LIPUS) application [9], [10] have been previously reported. Given the safety profile of LIPUS, efforts have been made to understand its bio-stimulatory effects, including the osteoblastic upregulation of IL-8, basic-FGF, VEGF, TGF-β, alkaline phosphatase, and the non-collagenous bone proteins [11], [12], [13], [14], while concomitantly down-regulating the osteoclastic response [11], [12] in cell-culture experiments. In particular, the acceleration of bone regeneration by LIPUS treatment has been the focus of recent studies. Wijdicks *et al.* [15] reported the osteoinductive response induced by rhBMP-2 is enhanced via LIPUS treatments, resulting in accelerated ectopic bone formation in a rat model. In the field of dentistry, LIPUS stimulation has been utilized for several types of interventions, such as the acceleration of osteogenic differentiation of the rat clonal cell line ROS 17/2.8 by increasing the expression of BMPs [16], enhancement of bone remodeling during the consolidation stage of distraction osteogenesis [17] or enhancement of the proliferation and differentiation of cementoblast lineage cells [18]. However, little information is available concerning the effects of LIPUS stimulation on alveolar bone remodeling during orthodontic tooth movement.

Tooth movement is closely related to the response to applied orthodontic forces that cause remodeling of periodontal tissues, especially alveolar bone. LIPUS stimulation has been reported to enhance fracture healing, to treat nonunion, and to accelerate bone maturation and remodeling during the consolidation stage of distraction osteogenesis, but the underlying mechanisms remain unclear. LIPUS therapy is a recently developed method for application of mechanical stress, and is used clinically to promote bone fracture healing [19], [20]. The stimulatory effects of LIPUS treatment on gene expression in rat bone marrow stromal cells have been reported [14]. Moreover, El-Bialy, *et al*. [21] demonstrated that LIPUS enhances mandibular growth in juvenile baboons, especially when combined with anterior mandibular jumping appliances. In addition, they found that LIPUS enhanced the healing process of orthodontically induced tooth-root resorption in humans [22].

Bone remodeling is regulated by local factors and certain key systemic hormones. Bone morphogenetic proteins (BMPs) are multifunctional growth factors within the transforming growth factor β (TGF-β) super family that were identified based on their ability to initiate ectopic bone and cartilage formation in adult animals [23], [24], [25]. After secretion and cleavage of a longer pre-protein, BMPs can bind to the extracellular matrix, their antagonists, co-receptors or transmembrane serine/threonine kinase receptors, which results in transcriptional and nontranscriptional responses [26]. Multiple studies have suggested that periodontal regeneration is mediated, at least in part, by bone morphogenetic protein-2 (BMP-2), and have demonstrated that BMPs induce the expression of osteogenic proteins and promote the regeneration of bone and periodontal tissues, including cementum [27], [28], [29].

Hepatocyte growth factor (HGF) is a heterodimeric molecule composed of an alpha and beta chain, which has been demonstrated to stimulate osteoblast proliferation [30] and modulate osteoblastic bone formation [31], [32]. HGF-mediated bone formation may involve activation of Runx2 [32]. However, the effect of HGF on BMP-2 expression in human alveolar bone remodeling is mostly unknown. We hypothesized that HGF regulates BMP-2 expression during alveolar bone remodeling. This study was designed to test this hypothesis as well as determine the underlying signaling pathways. Under LIPUS stimulation, we found that HGF, Runx2, and BMP-2 expression were upregulated during alveolar bone remodeling. In addition, Runx2 signaling pathways may be involved in the increase of BMP-2 expression which follows LIPUS stimulation.

The receptor activator of NFκB ligand (RANKL) is one of the key regulatory molecules in osteoclast formation and RANKL is involved in alveolar bone remodeling during orthodontic tooth movement, which is an important determinant regulating balanced alveolar bone resorption [33], [34], [35]. There is limited information regarding the effects of LIPUS stimulation on HGF/Runx2/BMP-2 signaling and RANKL as well, and whether these effects are responsible for the accelerated alveolar bone remodeling observed after such an intervention. The present study was designed to examine these effects during experimental tooth movement in rats.

## Materials and Methods

### Animals, antibodies and materials

All animal handling and surgical procedures were conducted strictly according to the guiding principles for the use of laboratory animals. This study was approved by the Animal Care Committee guidelines of the Fourth Military Medical University, Xi'an, China (Permit number: SCXK 2007–007).

Male Wistar rats (weighing 300±20 g, 12–13 weeks, n = 48 total) were randomized into control and LIPUS groups, and each group contained 6 subgroups (day 0, day 1, day 3, day 5, day 7, and day 14) (n = 8 per each group). They were maintained under controlled temperature (22±2°C), light dark periods of 12 hrs and with free access to water and commercial diet. All rats were acclimatized for 1 week before beginning the experiments. Tissue specimens (control and LIPUS groups) of 48 rats with first molars (both sides) and their periodontium were collected after verification by a pathologist, and immediately frozen in liquid nitrogen.

Primary antibodies against BMP-2 and RANKL were purchased from Abcam (ab14933 and ab45039, Cambridge, UK). Primary α-tubulin antibody was purchased from Millipore (MAB5566, Billerica, MA, USA); anti-β-actin (N-21), anti-HGF (H-170), and anti-Runx2 (S-19) primary antibodies were purchased from Santa Cruz Biotechnology (Santa Cruz, CA). Recombinant human HGF was purchased from PeproTech (Rocky Hill, NJ). The small interfering RNAs against Runx2 (sc-37145), and a control siRNA (sc-37007) were purchased from Santa Cruz Biotechnology (Santa Cruz, CA). FBS/Media (DMEM) were purchased from Invitrogen (Karlsruhe, Germany), SYBR Green PCR Master Mix for qRT-PCR was obtained from TaKaRa (Kyoto, Japan). Oligonucleotides (Table 1) were purchased from Sangon (Shanghai, China).

**Table 1:** Oligodeoxynucleotide primers used for qRT-PCR.

Gene	Primers	Product size (bp)
β-actin (human)	5′: -CACCACACCTTCTACAATGAG-3′ (F)	251
	5′: -GCATACCCCTCGTAGATGGGC-3′ (R)	
BMP-2 (human)	5′: -CTACATGCTAGACCTGTATCGC-3′ (F)	148
	5′: -CCCACTCGTTTCTGGTAGTTC-3′ (R)	
Runx2 (Rat)	5′: -ATGGCCGGGAATGATGAGAA-3′ (F)	151
	5′: -TCTGTCTGTGCCTTCTTGGT-3′ (R)	
BMP-2 (Rat)	5′: -CTATATGCTCGACCTGTACCG-3′ (F)	146
	5′: -CACTCATTTCTGAAAGTTCCTCG-3′ (R)	
HGF (Rat)	5′: -TGCCCTATTTCCCGTTGTG-3′ (F)	140
	5′: -CTAACCATCCACCCTACTGTTG-3′ (R)	
RANKL	5′: -AGAGCGCAGATGGATCCTAA-3′ (F)	180
	5′: -TTCCTTTTGCACAGCTCCTT-′ (R)	

### Experimental tooth movement

**Figure 1:**
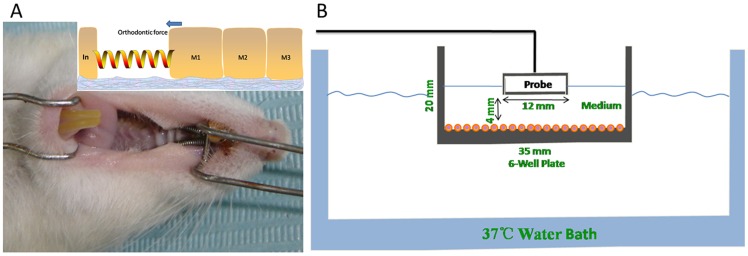
The methodology for tooth movement and a schematic diagram of LIPUS assembly for cell treatment. (A). View of rat oral cavity, showing that the upper first molar was moved mesially by a closed coil spring at 0.1N of orthodontic force. (B). The 6 well plate filled with medium was placed in the LIPUS field at a distance of 4 mm, which can generate optimized beam uniformity across the target cell region. The sterilized LIPUS transducer probe was suspended above the culture medium (using a clamp stand) partially immersed in the culture medium. The water bath was maintained at 37°C.

Orthodontic appliances were placed on the rats between the homolateral first upper molar and upper central incisor (Fig. 1A) under general anesthesia with xylazine (5 mg/kg, i.m.) and ketamine (100 mg/kg, i.m.). Experimental tooth movement was performed using a method previously described [36], in which a closed-coil spring (inner diameter: 0.030”, 3 M Unitek, CA, USA) was ligated to the maxillary first molar cleat with a stainless steel ligature wire (wire size: 20#, OSU, Hangzhou, China). The other side of the coil spring was ligated with the holes in the maxillary incisors drilled laterally just above the gingival papilla with a round bur (#1/4) using the same ligature wire. The upper first molar was moved mesially by the closed coil spring at a force of 0.1 Newton. The force was determined based on a preliminary study that showed that the upper first molar could be moved by orthodontic force without severe resorption [37], and the experiments were performed for a period of 14 days.

### LIPUS stimulation and temperature measurement

Animals were treated daily for different intervals (0 d, 1 d, 3 d, 5 d, 7 d, and 14 d) starting 0 d after orthodontic tooth movement, using 3% isoflurane gas (Surgivet/Anesco Isotec 4) as an anesthetic. Hair was shaved frequently for both groups to allow for consistent transducer placement. Ultrasonic coupling gel (Aquasonic 100, NJ, USA) was placed on the skin overlying the first molar sites and LIPUS was administered using a Exogen 2000 device (Smith & Nephew, Inc. London, UK). The LIPUS signal consisted of a 1.5-MHz frequency sine wave delivered in bursts lasting 200 μs, followed by a pause of 800 μs. The intensity output was 30 mW/cm^2^, which is the output signal of devices approved for clinical use. Meanwhile, the temperature rise of gingival surface of rat upper first molar was measured by pocket temperature laser infrared thermometer (S-HW1150, Century Harvest Electronics Co., Shenzhen, Guangdong, China) at different time points. These stimulation conditions were determined by the results from previous experiments [15]. The LIPUS treatment duration was 20 min each day, which is the FDA-approved clinical treatment used for nonunion fractures. According to previous study on a rat fracture model, the estimated amount of ultrasound intensity reaching the bone is 29.1 mW/cm^2^ for 30 mW/cm^2^ [38]. In our study, the LIPUS attenuation was negligible, so the above assumption was made. Sham LIPUS rats were handled the same as LIPUS rats, except the power to the LIPUS generator was not turned on.

The cells were subjected to LIPUS with modifications, as reported previously [16], [39], [40]. The exposure assembly is shown in Fig. 1B. A 6-well plate was held in place and the distance between the sterilized transducer probe (Asahi Irika Co, Ltd, Saitama, Japan) and cells was 4 mm, generating 1.5-MHz LIPUS in a pulsed-wave mode at 30 mW/cm^2^. The LIPUS exposure assembly was maintained at 37°C during all experiments. LIPUS stimulation was performed for 20 min daily for up to 14 days, when the cells reached confluence. The culture medium was replaced with fresh medium every 3 days. Control group cells were treated in the same manner without LIPUS stimulation.

### Measurement of tooth movement

To determine the distance of tooth movement, plaster models of the maxillae were made using silicone impression material (Heraeus, Hanau, Germany) before (0 d) and after initiating tooth movement (1 d, 3 d, 5 d, 7 d, and 14 d). The plaster models were measured using a vernier caliper (WR100, WIXEY, CA, USA) by setting the plane to pass through two points, which were the displacement between the first molar central fossa and second molar mesial surface to determine tooth movement.

### Tissue preparation

The experimental periods were set at day 0, 1, 3, 5, 7, and 14 (total 14 days) after start of tooth movement. After tooth movement, 4 rats of each group were perfused with 4% paraformaldehyde fixative (0.1 M phosphate buffer) for 2 hours through the ascending aorta under deep anesthesia, and the other rats in each group were used for qRT-PCR and Western blot detection (0 d, 3 d, and 7 d). The maxillae were resected and trimmed around the upper first molars. The trimmed tissues were decalcified by immersion in 5 percent ethylenediamine tetraacetic acid (EDTA)-2Na (7 percent sucrose, pH 7.4, 4°C) for 10 days, and the decalcified specimens were dehydrated through an ethanol series and embedded in paraffin. The samples that included the maximal tension region were sectioned with 5 μm continuous sections in the perpendicular direction by a Rotary microtome (RM2125RT, Leica, Heidelberger, Germany).

### Immunohistochemistry for BMP-2

The sections were deparaffinized in xylene and rehydrated through a graduated alcohol series. Endogenous peroxidase activity was blocked with 3% H_2_O_2_ in methyl alcohol. Antigens were retrieved in 10 mM sodium citrate (pH 6.0) by incubating at 92–98°C for 15 min. Nonspecific binding was blocked by incubating the sections with 0.1 M PBS containing 0.6% Triton X-100 and 10% normal goat serum for 1 hr at room temperature. After removal of excess blocking reagent, sections were incubated with rabbit anti-BMP-2 (1∶400) in 0.01 M PBS containing 1% bovine serum albumin (BSA) and 0.3% Triton X-100 at 4°C overnight. For negative controls, sections were incubated with blocking solution instead of primary antibody. After incubation with the primary antibody, sections were further incubated first with biotin-conjugated goat anti-rabbit IgG and subsequently with extravidin-peroxidase (1∶200, Sigma-Aldrich, MO, US) at room temperature for 1 hr, according to the manufacturer's instructions. Antibody complexes were visualized by development with 0.1% 3-diaminobenzidine in PBS with 0.03% H_2_O_2_ for 5 min at room temperature. Each step was followed by three washes in 0.01 M PBS. Sections were dehydrated through a graded ethanol series, cleared with xylene, and mounted with neutral resins. Photomicrographs were taken using an AxioImager M1 light microscope (Zeiss, Oberkochen, Germany). To evaluate the effect of LIPUS stimulation on the acceleration of alveolar bone remodeling, the number of BMP-2 positive cells in periodontal tissue were counted in the LIPUS stimulation and control groups, derived from four random fields in each slide (3 slides in each animal) for each treatment group.

### Cell culture

Human periodontal ligament (hPDL) cells were obtained from periodontally healthy donors, who had undergone extraction of teeth for orthodontic reasons, and were well identified (Fig. S1), according to the protocol used in a previous study [41]. Approval from the Ethics Committee of the Fourth Military Medical University and informed parental written consent were obtained (Approval ID: 2010–065). Cells dissected from the middle-third portion of the roots were grown in Dulbecco's modified Eagle medium (DMEM, Invitrogen, Karlsruhe, Germany) supplemented with 10% fetal bovine serum (FBS, Invitrogen), 100 units of penicillin and 100 μg/mL of streptomycin (Biochrom, Berlin, Germany). Cells from passage 5 were seeded (5×10^4^ cells/well) on Poly-D-Lysine Cellwares (Bection Dickinson, Bedford, UK) for 14 days. The cells were cultured at 37°C and higher temperatures (38°C, or 39°C) according to the experimental requirement in a humidified atmosphere of 95% air and 5% CO_2_, and the medium was changed every 3 days.

### Cell proliferation

hPDL cells were seeded at 1.5×10^3^ per well in 2 ml DMEM supplemented with 10% FBS containing 100 units of penicillin and 100 μg/mL of streptomycin. After 0, 1, 3, 5, 7, and 14 days, the attached cells were removed from the flasks by trypsinization to produce a single-cell suspension. The cells were counted with a L-BC3 differential counter (UNICO, Dayton, NJ, USA), and the number was defined at day 0, day 1, day 3, day 5, day 7, and day 14. At day 0, the flasks were filled with prewarmed DMEM, and the cell culture received 20 minutes of LIPUS exposure per day (the same parameters as for the animal study, Fig. 1B) for 14 days. The cell numbers were counted at day 0, day 1, day 3, day 5, day 7, and day 14 after LIPUS exposures. Cells with sham exposure served as the control and were counted in the same manner.

### qRT-PCR

Total RNA from hPDL cells and rat upper first molar periodontal tissue samples (n = 4) were extracted using Trizol reagent (Invitrogen, California, USA), and converted to cDNA using a PrimeScript RT reagent Kit (Takara Co., Shiga, Japan). qRT-PCR amplification was performed in a Real-Time PCR System (Applied Biosystems 7500, ABI). The samples were denatured at 95°C for 5 s, and then the primers were annealed at 60°C for 40 cycles at 30 s each. The primer sequences and PCR product sizes are shown in Table 1. *BMP-2*, HGF, RANKL, and Runx2 mRNAs were quantified by SYBR Green qRT-PCR and normalized to β-actin (Sangon, Shanghai, China). To ensure specificity of the PCR product amplification, the melting curves for standard and sample products were analyzed. All qRT-PCR reactions were performed three times.

### Western blot

hPDL cells were seeded 5×10^4^ cells/well in 6-well plates and cultured for up to 14 days in the presence and absence of daily LIPUS stimulation. The cells were harvested at each time point, washed once in phosphate-buffered saline (PBS) and lysed in RIPA lysis buffer. The periodontal tissue samples from rat upper first molars in LIPUS and control groups (n = 4) were cut into small pieces and homogenized in 100 μl lysis buffer containing protease inhibitors (Sigma). Protein concentration was determined by BCA (Pierce, Rockford, IL, USA). Aliquots (40 μg) were separated on a 12% SDS-PAGE and transferred to a PVDF membrane. The membrane was incubated with the specified primary antibody followed by peroxidase-conjugated secondary antibodies (goat anti-rabbit IgG) according to the manufacturer's protocol. An enhanced chemiluminescence (ECL) detection solution was applied (Amersham, Buckinghamshire, UK). The relative protein levels in different cell lines were normalized to the signal intensity of α-tubulin/β-actin as an internal control. Western blots were analyzed by densitometry using the NIH-ImageJ program (http://rsb.info.nih.gov/nih-image/), which is publicly available through the National Institute of Health.

### Statistical analysis

All experiments were performed in triplicate and standard deviations were calculated. The statistics were performed using SPSS V13.0 (Chicago, IL). Differences/correlations between groups were calculated using a Student's *t*-test or Pearson-test. A *P* value of less than 0.05 was considered statistically significant.

## Results

All rats remained healthy during the entire study period, and food and water intake appeared unaffected by the orthodontic appliances. Except for a short period following surgery, there was an overall gain in weight throughout the lifetime of the rats.

### Orthodontic tooth movement and temperature rise by LIPUS stimulation

**Figure 2:**
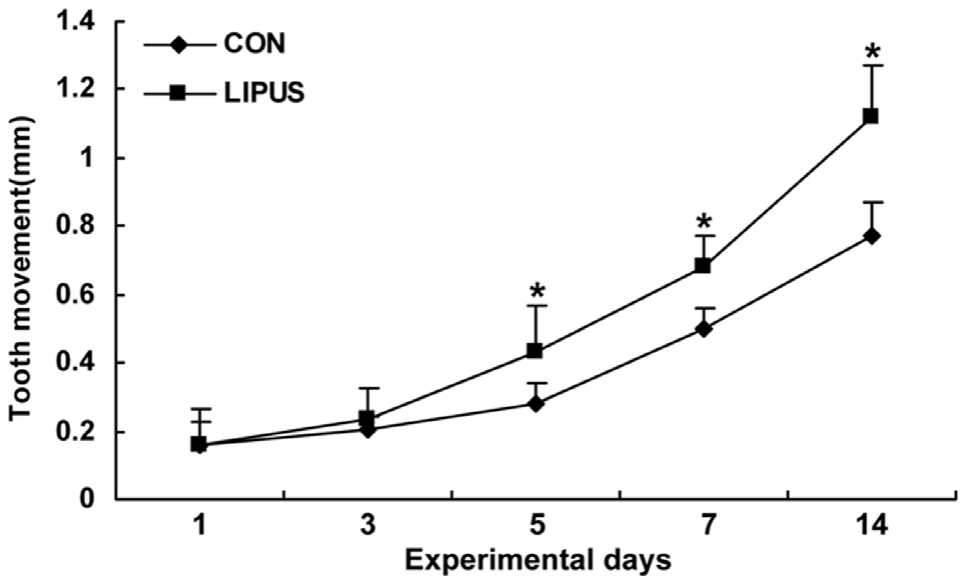
The effect of LIPUS stimulation on rat tooth movement. The amount of tooth movement in the LIPUS group was significantly greater than the control group on day 5, day 7, and day 14. *Significantly different from corresponding non-stimulation group (*P<0.05*). Values are shown as the mean ± SD, and n = 6.

The amount of tooth movement was significantly greater in the stimulation group on day 5, 7 and 14, compared with the non-stimulation groups (Fig. 2). In the control group, the amount of molar movement has a mean of 281 μm by day 5 (range 218–344 μm), and the amount of molar movement has a mean of 497 μm by day 7 (range 436–558 μm). In LIPUS stimulation groups, the amount of tooth movement increased by approximately 55 percent and 37 percent by day 5 and day 7, respectively (LIPUS group by day 5: mean 435 μm, range 303–567 μm; LIPUS group by day 7: mean 681 μm, range 590–772 μm). Overall, in the control groups, there was a continuously increasing movement of the molars from days 3 to 14, ending with a mean of 773 μm (range 678–868 μm). Following the application of LIPUS, the amount of tooth movement after 14 days increased by approximately 45 percent (LIPUS group: mean 1118 μm, range 965–1271 μm). The temperature rise of the gingival surface of upper first molars by pocket temperature laser infrared thermometer at different time points (0 min, 4 min, 8 min, 12 min, 16 min, and 20 min) was measured in LIPUS and control groups (Fig. S2). At these time points, the temperature rise was 0.96°C, 1.71°C, 2.23°C, 2.52°C, and 2.66 °C in LIPUS groups respectively, compared with control groups, and the peak of temperature rise appeared at 20 min.

### LIPUS enhances BMP-2 expression

**Figure 3:**
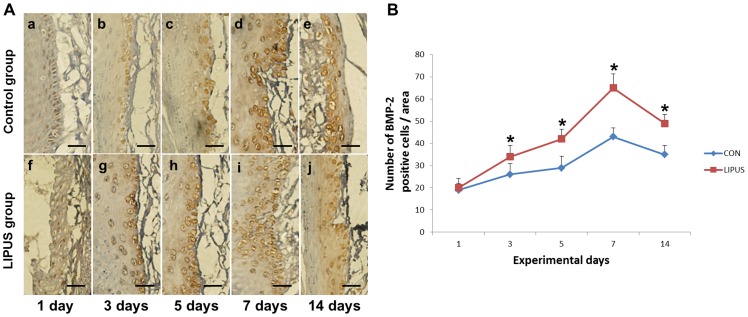
Quantitative analysis of BMP-2 expression. (A). Effects of LIPUS stimulation on BMP-2 positive cells in the periodontium is shown by immunohistochemistry (bar: 20 μm). BMP-2 immunoreactivity was observed on the alveolar bone surface in the periodontium of the tension side in the LIPUS stimulation group (g–j) on days 3, 5, 7, and 14, significantly higher than that (b–e) in the non-stimulation group. (B). The number of BMP-2 positive cells in the LIPUS stimulation group was greater than that in the control group on days 3, 5, 7, and 14. *indicates *P<0.05*. Values are shown as the mean ± SD, and n = 4.

There were no obvious side effects of LIPUS stimulation on the periodontium, and HE stain is shown in light microscope images (Fig. S3). At 1 day after the start of tooth movement, a few BMP-2 positive PDL cells were observed on the surfaces of the alveolar bone [Fig. 3A (a, f)]. At days 3, 5, and 7 after the start of tooth movement, many resorption lacunae with BMP-2 positive hPDL cells appeared on the alveolar bone tension surface side [Fig. 3A (b–e, g–j)]. The number of BMP-2 positive cells in the LIPUS stimulation group was greater than that in the non-stimulation group. Fourteen days after the initiation of tooth movement, regenerated bone with BMP-2 positive hPDL cells were recognized on the surface of the alveolar bone tension surface side [Fig. 3A (e, j)]. In our quantitative evaluation, the number of BMP-2 positive cells was found to be significantly increased in the LIPUS stimulation group on days 3, 5, 7 and 14 compared with the non-stimulation group.

### LIPUS stimulation has no effect on cell viability and the elevation of temperature has no effect on BMP-2 expression

The hPDL cell viability in the presence and absence of daily LIPUS stimulation was measured. LIPUS stimulation for up to 14 days did not affect cell viability (Fig. S4 A). To further evaluate whether the thermal effect of the LIPUS has any effects on BMP-2 expression in hPDL cells, the cells were incubated at different temperatures (37°C, 38°C, 39°C), and BMP-2 mRNA and protein amounts were detected by qRT-PCR and Western blot, respectively. It indicated that no significant difference in high temperature groups (38°C, 39°C), compared with control group (37°C) was observed (Fig. S4 B–D).

### LIPUS stimulation increases osteoclast number and RANKL expression on the pressure side

**Figure 4:**
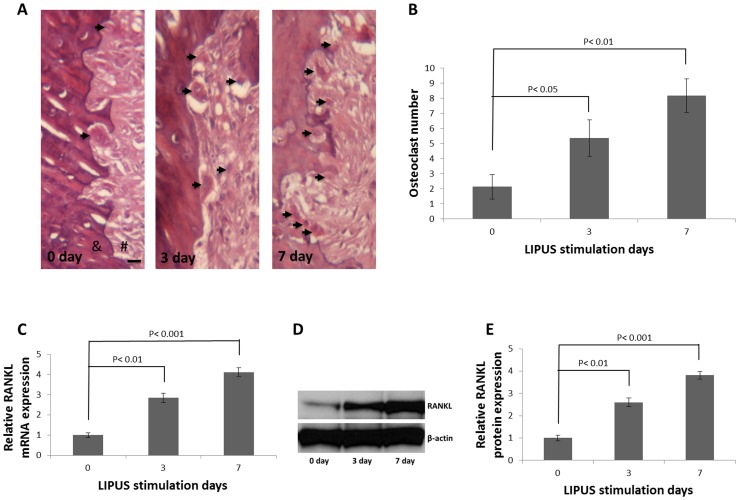
LIPUS stimulation increased osteoclast number and RANKL expression on the pressure side. (A). Light micrographs of PDL tissue on the compressed side in rat on days 0, 3, and 7 after LIPUS stimulation. A few osteoclasts (arrowheads) are seen in the PDL proper on day 3 and day 7. #: PDL; &: alveolar bone, scale bar, 20 μm. (B). Osteoclast number was counted in the area indicated in Fig. 4A. The quantified data shown represent the mean ± SD for three rats, and each rat contains four random fields, and all the osteoclasts were verified by a pathologist. LIPUS stimulation 0 day vs LIPUS stimulation 3 day, *P<0.05;* 0 day vs 7 day, *P<0.01.* Rat upper first molars were stimulated with or without LIPUS for different time intervals, and RANKL mRNA (C) and protein amount (D, E) increased at 3 and 7 days after LIPUS stimulation than day 0. LIPUS stimulation 0 day vs LIPUS stimulation 3 day, *P<0.01;* 0 day vs 7 day, *P<0.001.*

Bone remodeling is regulated by the rate limiting step during tooth movement, which occurs on the pressure side and is associated with bone resorption. It is important to demonstrate whether there are changes in osteoclast numbers and RANKL expression in the pressure side. Therefore, we evaluated the osteoclast numbers and RANKL expression at different time points after LIPUS stimulation. The HE staining data showed that, compared with control group, LIPUS stimulation significantly increased osteoclast number, especially on day 7 (Fig. 4A, B; *P<0.01*), and the qRT-PCR (Fig. 4C) and Western blot data (Fig. 4D–E) showed that LIPUS stimulation significantly increased RANKL expression, especially on day 7 (*P<0.001*).

### HGF and Runx2 are involved in the action of BMP-2 under LIPUS stimulation

**Figure 5:**
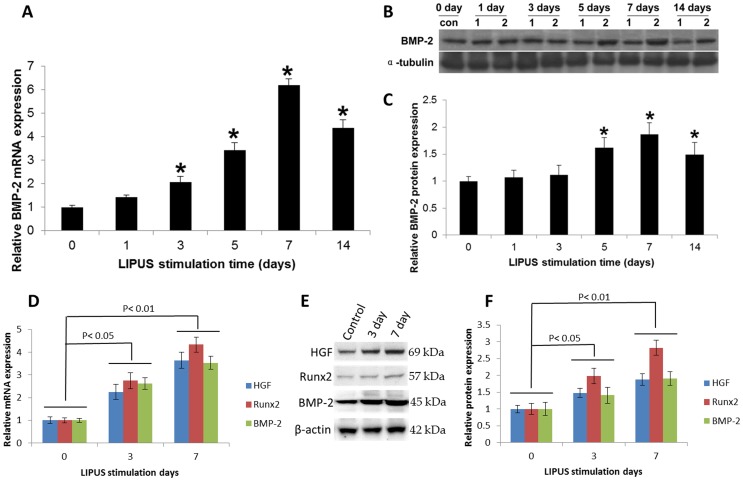
LIPUS stimulation enhanced the BMP-2 signaling pathway gene expression *in vitro* and *in vivo*. (A). hPDL cells were cultured in the presence and absence of daily LIPUS stimulation. BMP-2 mRNA expression was determined using qRT-PCR. (B). BMP-2 protein (ROW 1; 45 kDa) can be detected in the control and LIPUS groups (1: CON; 2: LIPUS). The LIPUS groups showed higher expression of BMP-2 from day 5. (C). Quantification of BMP-2 protein expression in the LIPUS stimulation group was greater than that in the control group on days 5, 7, and 14. *indicates *P<0.05*. (D–F). Rat upper first molars were stimulated with or without LIPUS for different time intervals, and HGF, Runx2, BMP-2 were determined by qRT-PCR and Western blot, respectively. The data indicated that LIPUS increased HGF, Runx2, and BMP-2 mRNA (D: LIPUS stimulation 0 day vs LIPUS stimulation 3 day, *P<0.05;* 0 day vs 7 day, *P<0.01*) and protein expression (E, F: LIPUS stimulation 0 day vs LIPUS stimulation 3 day, *P<0.05;* 0 day vs 7 day, *P<0.01*) *in vivo*. The data are the mean ± SD of three separate experiments.

The effect of LIPUS stimulation on the expression of mRNAs encoding BMP-2 in hPDL cells was determined by qRT-PCR. Compared with control cells, LIPUS-treated cells expressed significantly more BMP-2 mRNA on days 3–14 of culture; the levels peaked at day 7 and remained elevated through day 14 (Fig. 5A). BMP-2 protein expression in human hPDL cells both non-treated and treated with LIPUS was investigated using Western blot analysis. At days 5, 7, and 14 of LIPUS stimulation, the amount of BMP-2 protein (Fig. 5B, C) was substantially increased compared with the control group (*P<0.05*). To determine whether HGF and Runx2 were involved in LIPUS triggered BMP-2 expression *in vivo*, the HGF, Runx2, and BMP-2 expression level of LIPUS and control tissue groups were detected by qRT-PCR and Western blot. Compared with control groups, LIPUS-treated groups expressed significantly more HGF, Runx2, and BMP-2 mRNA (Fig. 5D) and protein (Fig. 5E, and 5F) on day 3 (*P<0.05*) and day 7 (*P<0.01*) of stimulation during the rat tooth movement.

### HGF mediated BMP-2 expression requires Runx2

**Figure 6:**
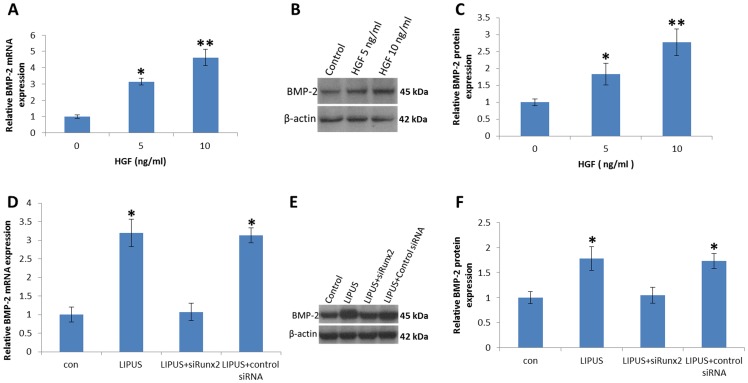
LIPUS stimulation increased BMP-2 expression mediated by Runx2. (A). hPDL cells were incubated with HGF for 24 h, and BMP-2 mRNA was examined by qRT-PCR. (B and C) hPDL cells were incubated with HGF for 48 h, and BMP-2 protein amounts were detected by Western blotting. The data shows that HGF significantly increased BMP-2 expression. (D). Cells were transfected with Runx2 siRNA for 24 h followed by stimulation with LIPUS for 5 days, and BMP-2 mRNA expression was examined by qRT-PCR. (E and F) hPDL cells were transfected with Runx2 siRNA for 5 days, and BMP-2 protein expression was examined by Western blot. Transfection of cells with Runx2 siRNA reduced LIPUS-increased BMP-2 expression. *P<0.05 (**P<0.01) as compared with the control group.

Previous study has shown that Runx2 is involved in HGF-mediated BMP-2 production in osteoblasts [32]. To examine the effects of HGF on BMP-2 expression, hPDL cells were exposed to HGF during LIPUS stimulation, and the mRNA and protein expression of BMP-2 was determined. Treatment of hPDL cells with HGF (5 ng/ml, and 10 ng/ml) for 24 h induced BMP-2 mRNA expression (Fig. 6A). In addition, stimulation of cells with HGF also led to increased BMP-2 protein expression by Western blotting (Fig. 6B and C). It has been reported that HGF exerts its effects through interaction with a key transcription factor Runx2 [32]. Pretreatment of hPDL cells with Runx2 siRNA reduced LIPUS-increased BMP-2 mRNA expression (Fig. 6D). In addition, transfection of cells with Runx2 siRNA also reduced LIPUS-increased BMP-2 protein expression (Fig. 6E and F). Therefore, an interaction between HGF and Runx2 is important for BMP-2 production in hPDL cells.

## Discussion

Thermal and non-thermal effects are involved in LIPUS biophysical actions, and previous studies have shown that LIPUS may promote wound healing and angiogenesis in periodontal tissue by non-thermal rather than by thermal effects [42]. Several studies have demonstrated that loading of mechanical stress onto bone causes osteoblasts to respond in various ways [43], such as by promoting BMP production [44]. Kim *et al.* investigated the combined effects of corticision and low level laser therapy to promote tooth movement rate and paradental remodeling in beagles [45]. We found that LIPUS stimulation can promote orthodontic tooth movement by upregulating HGF/Runx2/BMP-2 signaling pathway gene expression and RANKL expression. We also determined that LIPUS treatment significantly upregulates HGF gene expression starting from day 3, and HGF through Runx2 regulates BMP-2 gene expression.

LIPUS has many clinical advantages including the fact that it is a biological stimulus, easy to use, and noninvasive, and has been widely used in clinical medicine. The bone fracture healing process usually includes three periods: cartilage formation, endochondral ossification, and bone remodeling. Azuma *et al*. reported that LIPUS promoted fracture healing in all three periods by evaluating each process [46]. Katano *et al*. found that LIPUS exposure reduced the time to complete endochondral ossification from 4 weeks to 3 weeks after fracture. They reported that LIPUS accelerates fracture healing through promotion of endochondral ossification and bone remodeling [47]. El-Bialy, *et al*. clarified the possible mechanisms by which LIPUS minimizes orthodontically induced tooth root resorption by enhancing dentine and cementum deposition, which makes a preventive layer against root resorption in a mandible slice organ culture [48]. BMP expression is significantly increased by mechanical stress such as optimal compressive force and shear stress [44], [49]. Recent studies investigated the effects of daily LIPUS treatment, which increased expression of BMP-2 mRNA [50] and the expression profiles of BMPs [16]. We speculated that the effects of LIPUS treatment on tooth movement may be due to BMP-2 signaling upregulation.

We also observed osteoclast numbers and RANKL expression on the pressure side, and both of them increased significantly after LIPUS stimulation. Alveolar bone remodeling is an adaptive mechanism to better meet the various needs of the body, and in the orthodontic clinic, remodeling allows effective tooth movement and inhibition of relapse. Therefore, promotion of alveolar bone remodeling has important clinical significance.

In orthodontic treatment, tooth movement and alveolar bone remodeling accompany each other, so in animal experiments and clinical observations, the speed of tooth movement is used to reflect bone remodeling [37]. Orthodontic tooth movement and alveolar bone remodeling in the periodontal tissues under the effect of orthodontic force is a complex process and the result of interaction between many factors. BMP-2, as the osteoclast differentiation factor with support of other osteoclast cell differentiation factors, directly or indirectly leads to differentiation of osteoclasts [51]. LIPUS, through BMP-2 signaling, activates proliferation of osteoblasts and BMP-2 signaling certainly has a regulatory role during alveolar bone remodeling, but other factors may also promote alveolar bone remodeling.

The ultimate goal of orthodontic tooth movement is to move teeth in the most effective way and at the same time to reduce side-effects. In this study, the effect of LIPUS stimulation on experimentally induced tooth movement was investigated in the rat model based on the measurement of tooth movement distance, immunohistochemistry, Western blot and qRT-PCR techniques. Our data show that LIPUS stimulation promotes alveolar bone remodeling by enhancing the expression of BMP-2 signaling pathway, and RANKL expression in a rat orthodontic tooth movement model, and confirms that LIPUS upregulates BMP-2 gene expression through Runx2 in hPDL cells. Shortening the orthodontic treatment regimen has been an important research topic, and has drawn the attention of many investigators worldwide to explore the biology behind orthodontic tooth movement.

Although the absorption of the ultrasound signal may result in energy conversion to heat, this effect is extremely small for low frequency ultrasonic waves, well below 1°C [52]. In this study, we measured the temperature rise of the gingival surface of the upper first molar in LIPUS groups at different time points. After 20 minutes of stimulation, the temperature was highest (2.66°C higher than the temperature of control group), and temperature rise accompanied stimulation time, showing a similar parabolic growth. This is closely related to the maxillofacial region rich in blood circulation and the gingival surface heating, which was rapidly reduced by blood circulation. Moreover, the saliva in the oral cavity may also dissipate some heat from the gingival surface. In hPDL cells, the higher temperatures (38°C, 39°C) did not increase BMP-2 expression compared with the control group (37°C), indicating that the LIPUS upregulation of the BMP-2 signaling pathway was through its non-thermal effect.

We found that BMP-2 expression in the presence of daily LIPUS stimulation increased gradually from day 5 of culture, and the BMP-2 expression level at days 5, 7, and 14 was significantly higher in the LIPUS-treated cells than in the controls, indicating that daily LIPUS stimulation enhances BMP-2 gene expression in cultured hPDL cells. To confirm the above findings, we performed qRT-PCR analysis to assess the expression of BMP-2 mRNA. In cells cultured in the presence of LIPUS stimulation, we found that the levels of BMP-2 mRNA was significantly higher at days 5–14, peaking at day 7 and remaining nearly constant through day 14, providing evidence that BMP-2 acts as a transcription factor in LIPUS-stimulated hPDL cells. We also evacuated the HGF/Runx2/BMP-2 signaling pathway, and confirmed that LIPUS through Runx2 upregulated BMP-2 gene expression.

In conclusion, LIPUS stimulation enhanced orthodontic tooth movement via elevation of the HGF/Runx2/BMP-2 signaling pathway gene expression and RANKL expression in an orthodontic rat model. Furthermore, we confirmed the concept that BMP-2 mRNA and protein expression were enhanced via Runx2 expression in hPDL cells by LIPUS stimulation *in vitro*. Together, these findings suggest that LIPUS stimulation accelerates alveolar bone remodeling, thus potentially shortening the orthodontic treatment period in the clinic. Because LIPUS is safe and noninvasive, it would be a promising new adjuvant therapy for accelerating orthodontic tooth movement, which could result in shortening orthodontic treatment.

## Supporting Information

Figure S1 **The characteration of primary hPDL cells in culture.** (A) Primary culture of hPDL cells at the fourth passage (200×); the cells had elongated spindle-shape, with oval nucleus located in the central position. There were 2–4 cytoplasmic extensions. (B) hPDL cells were positive for vimentin staining (400×); cytoplasm was red, and positive signals were distributed evenly and the nucleus was blue. (C) hPDL cells were negative for cytokeratin staining by immunofluorescence (400×).
(TIF)

Figure S2 **The temperature rise in 20 min for the 1.5 MHz LIPUS described earlier.** The total acoustic density generated by the transducer in this case was 30 mW/cm^2^. Mean temperature rise ± SD is shown, and n = 3.
(TIF)

Figure S3 **Effects of LIPUS stimulation on the periodontium by light microscopy.** Effects of LIPUS stimulation on the periodontium is shown in light microscope images (HE, bar: 50 μm) (* molar root; # PDL; & alveolar bone). At 1 day after the start of tooth movement, the arrangement of fibers and fibroblasts became coarse and irregular, and blood capillaries shrank [Fig. S3 (b, h)]. At days 3, 5, and 7 after initiation of orthodontic tooth movement, the PDL was composed of a coarse arrangement of fibers and expanded blood capillaries, and new alveolar bone with osteoblasts also appeared on the alveolar bone tension surface side [Fig. S3 (c–f, i–l)].
(TIF)

Figure S4 **Effect of LIPUS on cell viability and elevation of temperature on BMP-2 expression.** (A) hPDL cells were cultured in the presence and absence of daily LIPUS stimulation and the cell numbers were determined at day 1, 3, 5, 7, and 14 of culture. Significant differences were not observed between LIPUS stimulation and non-stimulation groups at any observed time points. The higher temperatures do not affect BMP-2 mRNA (B) or protein amounts (C, D), compared with normal culture temperature. The data are shown as the mean ± SD of three separate experiments.
(TIF)
