# Supplementary figures and images for: Low-Intensity Pulsed Ultrasound Accelerates Tooth Movement via Activation of the BMP-2 Signaling Pathway
Source: PLoS One. 2013 Jul 23;8(7):e68926. doi: 10.1371/journal.pone.0068926 (PMC3720872; doi:10.1371/journal.pone.0068926)

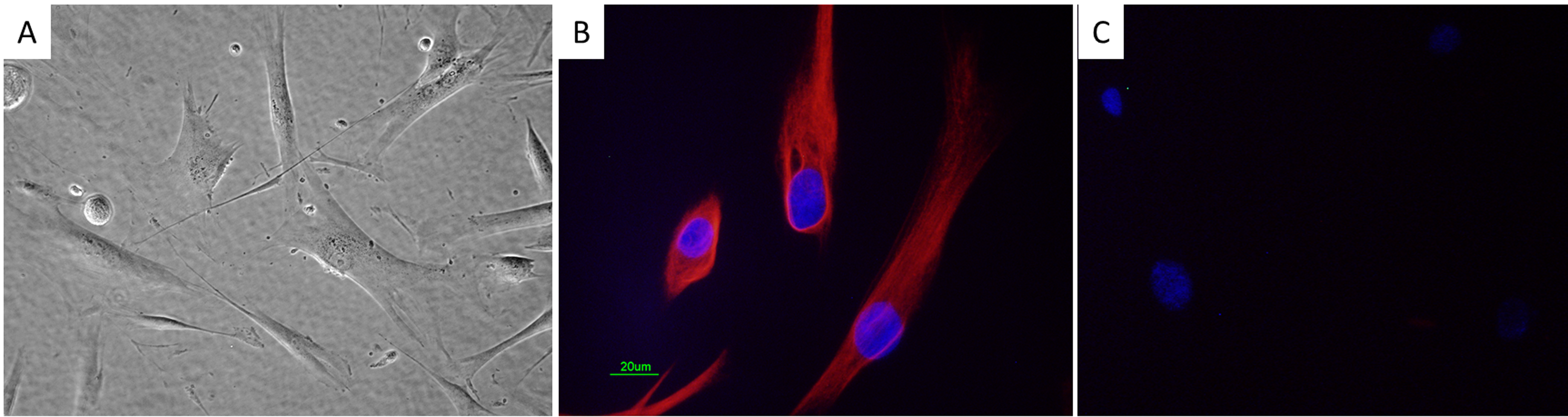

Supplement: Figure S1 — The characteration of primary hPDL cells in culture. (A) Primary culture of hPDL cells at the fourth passage (200×); the cells had elongated spindle-shape, with oval nucleus located in the central position. There were 2–4 cytoplasmic extensions. (B) hPDL cells were positive for vimentin staining (400×); cytoplasm was red, and positive signals were distributed evenly and the nucleus was blue. (C) hPDL cells were negative for cytokeratin staining by immunofluorescence (400×). (TIF) [file pone.0068926.s001.tif]

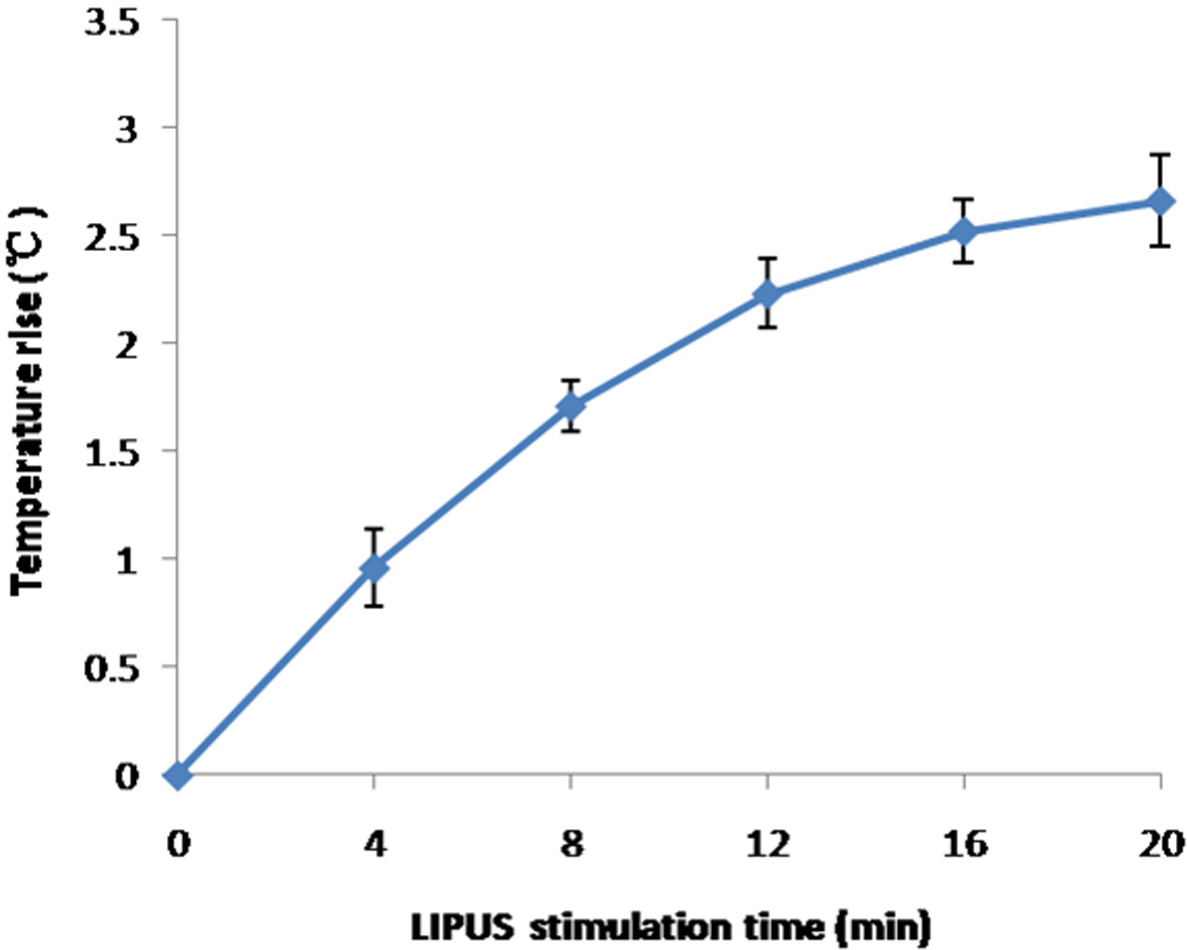

Supplement: Figure S2 — The temperature rise in 20 min for the 1.5 MHz LIPUS described earlier. The total acoustic density generated by the transducer in this case was 30 mW/cm2. Mean temperature rise ± SD is shown, and n = 3. (TIF) [file pone.0068926.s002.tif]

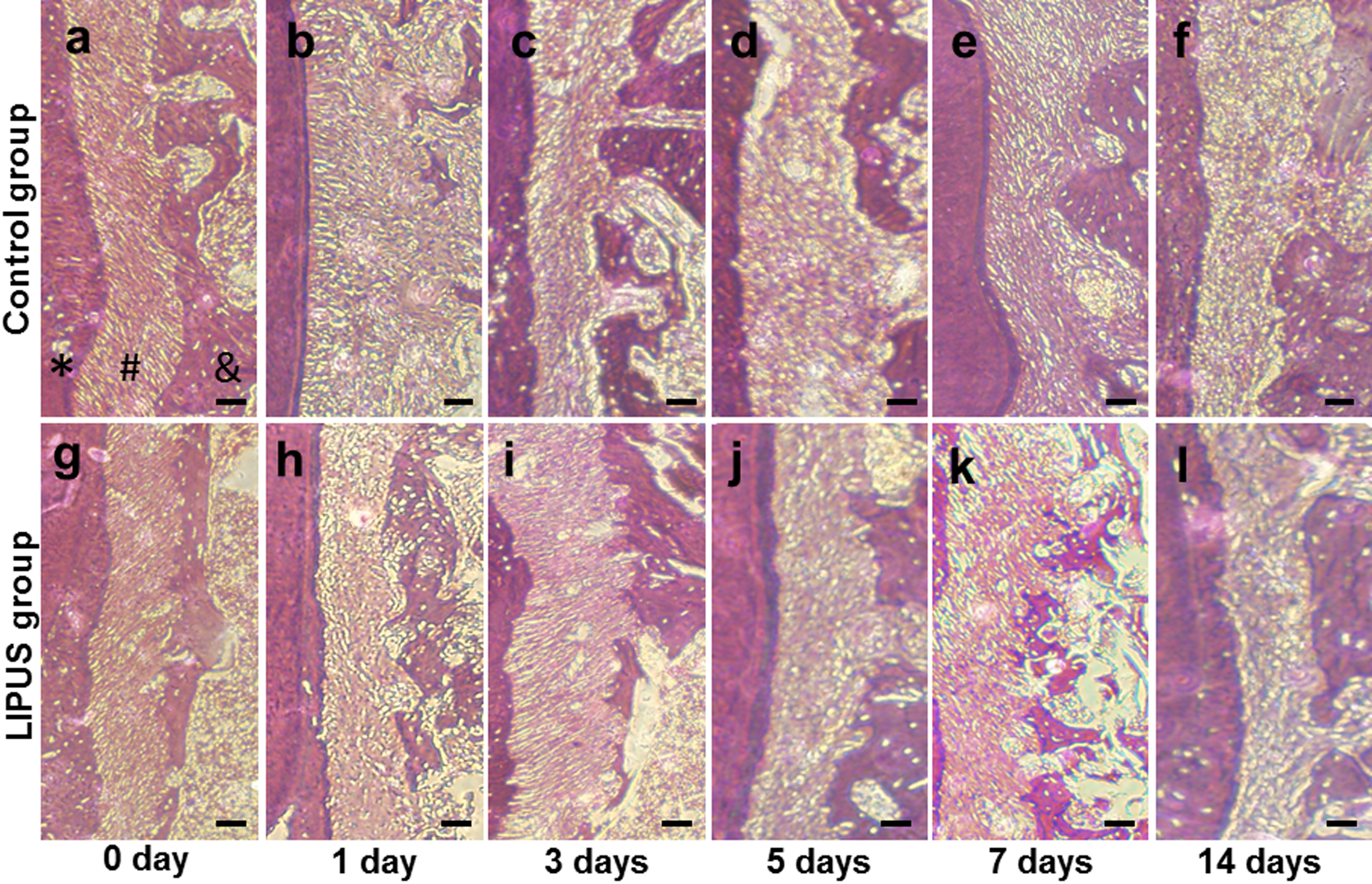

Supplement: Figure S3 — Effects of LIPUS stimulation on the periodontium by light microscopy. Effects of LIPUS stimulation on the periodontium is shown in light microscope images (HE, bar: 50 μm) (* molar root; # PDL; & alveolar bone). At 1 day after the start of tooth movement, the arrangement of fibers and fibroblasts became coarse and irregular, and blood capillaries shrank [Fig. S3 (b, h)]. At days 3, 5, and 7 after initiation of orthodontic tooth movement, the PDL was composed of a coarse arrangement of fibers and expanded blood capillaries, and new alveolar bone with osteoblasts also appeared on the alveolar bone tension surface side [Fig. S3 (c–f, i–l)]. (TIF) [file pone.0068926.s003.tif]

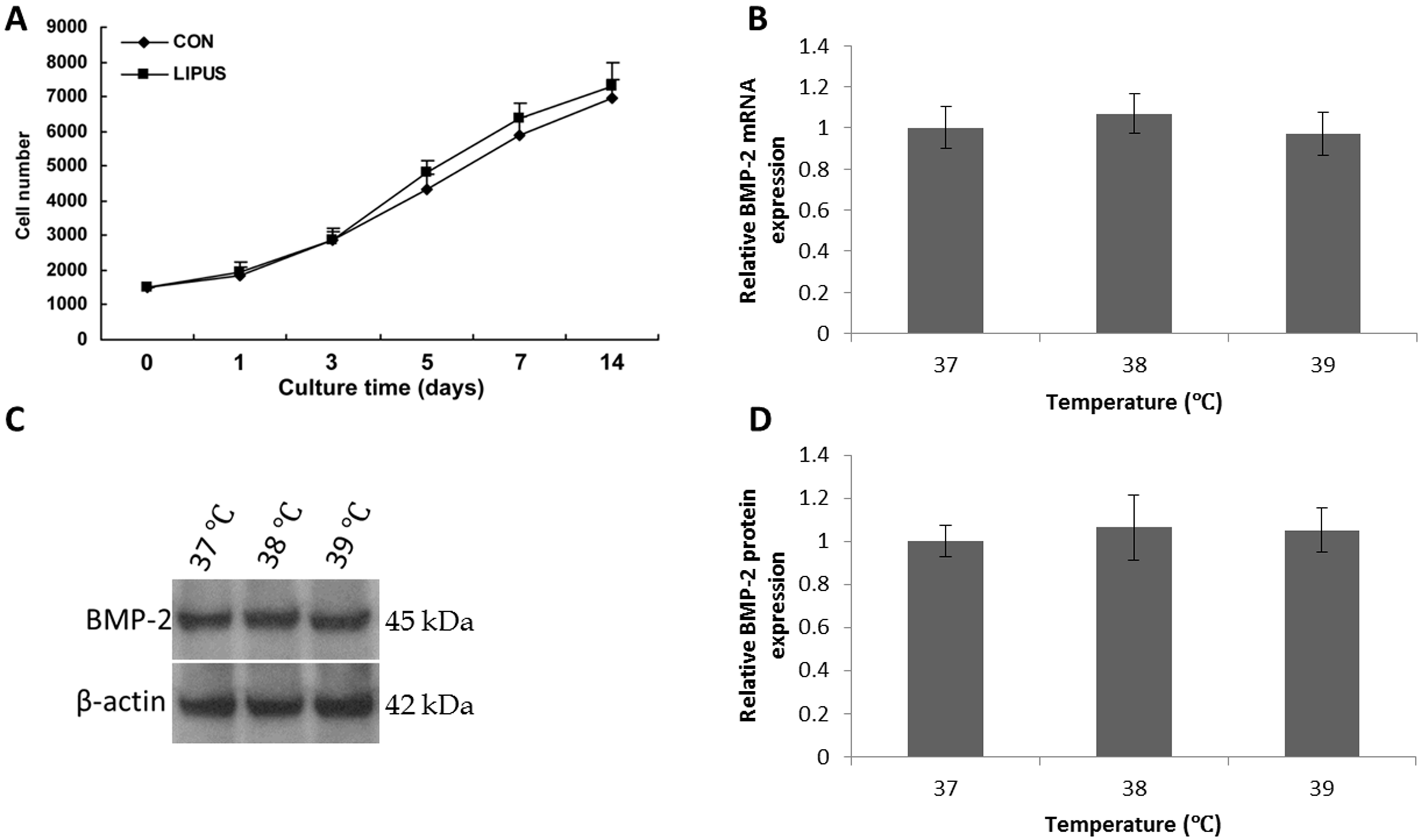

Supplement: Figure S4 — Effect of LIPUS on cell viability and elevation of temperature on BMP-2 expression. (A) hPDL cells were cultured in the presence and absence of daily LIPUS stimulation and the cell numbers were determined at day 1, 3, 5, 7, and 14 of culture. Significant differences were not observed between LIPUS stimulation and non-stimulation groups at any observed time points. The higher temperatures do not affect BMP-2 mRNA (B) or protein amounts (C, D), compared with normal culture temperature. The data are shown as the mean ± SD of three separate experiments. (TIF) [file pone.0068926.s004.tif]
